# Editorial: FMD Research: Bridging the Gaps With Novel Tools

**DOI:** 10.3389/fvets.2021.686141

**Published:** 2021-05-17

**Authors:** Alejandra V. Capozzo, Mariano Pérez-Filgueira, Wilna Vosloo, Cyril G. Gay

**Affiliations:** ^1^Instituto de Virología e Innovaciones Tecnológicas (IVIT), Centro de Investigaciones en Ciencias Veterinarias y Agronómicas (CICVyA), Instituto Nacional de Tecnología Agropecuaria (INTA)- Consejo Nacional de Investigaciones Científicas y Técnicas (CONICET), Buenos Aires, Argentina; ^2^Australian Centre for Disease Preparedness (Formerly Australian Animal Health Laboratory), Commonwealth Scientific and Industrial Research Organisation (CSIRO)-Health and Biosecurity, Geelong, VIC, Australia; ^3^Foreign Animal Disease Research Unit, Plum Island Animal Disease Center, Agricultural Research Service, United States Department of Agriculture, Greenport, NY, United States

**Keywords:** foot-and-mouth disease virus, transboundary diseases, research gaps, global research network, livestock industry, global foot-and-mouth disease control

Foot and mouth disease (FMD) remains a major threat for livestock industries, affecting large numbers of cloven-hoofed animal species worldwide with an estimated annual global economic loss of between US$6.5 and 21 billion and ~US$ 5 billion related to production losses and vaccination alone (1). The devastating effects of FMD affects all countries around the world impacting from smallholders' farms in low-income countries suffering reduced productivity (2), to middle-to-high income countries affected by the cost of prevention, surveillance, and control measures in domestic species as well as the severe restrictions imposed on international trade (3).

FMD's etiological agent is a small non-enveloped positive sense single-stranded RNA virus (FMDV) belonging to the *Picornaviridae* family, genus *Aphthovirus* (4). Considered as one of the most infectious amongst human or animal disease agents known, the virus is recognized for its high antigenic variability and efficient transmission among a wide range of susceptible animal species (5). Although progress in the global control of FMD is ongoing and supported by international organizations such as the World Organisation for Animal Health (OIE) and United Nations Food and Agriculture Organisation, the disease is still endemic in many parts of the world, circulating in over 75% of the global livestock population (2).

Scientists working on FMD research around the world are networking through the Global Foot and Mouth Disease Research Alliance (GFRA—https://www.ars.usda.gov/gfra/). The GFRA continually assesses research gaps and priorities, shares the latest scientific advances, and enables and promotes collaborations and networking among the different laboratories conducting FMD research worldwide. This Research Topic focuses on recent studies that address FMD knowledge gaps, comprising 24 original manuscripts covering priority areas such as diagnostics, field surveillance, evolution, molecular epidemiology, immunopathogenesis, vaccine development, immunology, and antiviral therapy.

Strategies for FMD control vary between regions depending on their epidemiological situation. To manage the risk, it is essential that governments, farmers, veterinarians, and industries engage in significant surveillance, prevention, control, and preparedness programs.

Field surveillance is a critical component of any disease control program. Singanallur et al. demonstrated circulation of FMDV in goat populations in Lao, using two serological tests on a set of samples collected from several provinces and analyze different factors related to seropositivity. Using a different strategy, Ularamu et al. collected tissue samples from 27 outbreaks of FMD in different states of Nigeria to gain more knowledge on FMDV circulation in this country. FMDV isolates obtained were serotyped and further characterized by VP1 sequencing and phylogenetic analysis. Velazquez-Salinas et al. provided a collection of VP (viral protein) 1 and P1 (complete capsid coding region) protein sequences from 29 different districts in Uganda, which combined with geographic information, may be used to perform phylogenetic analyses and antigenic characterization of the FMDV variants circulating in this region. Finally, aiming to improve the serotype-independent FMDV detection, Mishu et al. analyzed the comparative evolutionary divergence of VP2 and VP1 nucleotide sequences to determine the level of conservation in VP2 at different hierarchical levels of three FMDV serotypes (O, A, and Asia1) currently circulating in Asia.

For many years, FMD diagnostic tests have evolved to analyze different aspects of the disease and vaccination, however many of them still need to be assessed and validated for both sensitivity and specificity. Gray et al. analyzed the comparative performance for FMDV isolation between a highly sensitive primary cell culture (BTY) and two continuous cell lines derived from goat (ZZ-R 127) and swine (LFBK-αVβ6). Also, new approaches aim to improve the efficacy of the surveillance programs. A phage display library was explored by Chitray et al. to identify antigenic determinants for recombinant vaccines and for the generation of reagents for improved diagnostic enzyme-linked immunosorbent assays (ELISA) specific for the FMDV serotypes A, Southern-African Territories (SAT) 1 and SAT3. In a different approach, the article by Armson et al. discussed the use of pooled milk and rRT-PCR for active large-scale dairy farm surveillance. With this strategy, the authors provided evidence of subclinical virus infection in vaccinated herds that could be important in the epidemiology of FMD in endemic countries where vaccination is used. Also discussing the implementation of alternative surveillance strategies, Eschbaumer et al. reviewed benefits and limitations of empowering veterinarians to perform rapid diagnostic testing in the field. The need for point-of-care (pen-side) diagnostic test kits, such as lateral flow devices and mobile versions of RT-PCR and RT-LAMP, was also highlighted by Wong et al. in a review that analyzed some of the advances in FMD diagnostic tools.

Simultaneously, novel FMD vaccines and formulations are being developed. A new fully DIVA-compatible vaccine platform is presented, based on highly attenuated virus containing negative antigenic markers in conserved non-structural proteins to enable the differentiation of vaccinated from infected animals (Hardham et al.). Efforts are also moving toward strategies that eliminate growing live FMDV during the manufacturing process of vaccines. This topic includes two approaches: Cañas-Arranz et al., studied the antibody neutralizing and cellular immune responses elicited in swine by synthetic antigens based on dendrimer peptides harboring the major FMDV antigenic B-cell site and a T-cell epitope from 3D polymerase protein, while Mignaqui et al. reported on the optimization of the production strategies for virus like particle (VLP)-based FMD vaccine antigens generated by transient gene expression in mammalian cells. A comparative performance between VLP-based and conventional FMD vaccines on the humoral response and *ex-vivo* activation of dendritic cells in cattle is also presented by Quattrocchi et al.. Finally, Bidart et al. studied the use of particle adjuvants as immunostimulants in experimental vaccines formulated with inactivated whole FMDV antigens in mice and cattle.

In direct relation with the generation of novel prophylactic and therapeutic tools, fundamental studies on the immunological responses to the infection and vaccination can provide scientific bases for such developments. Marrero Diaz De Villegas et al. analyzed the feasibility of using computational methods based on the side chain optimizations to predict neutralizing interactions between antibodies and FMDV antigenic sites. Also using genetic and structural data, Maake et al. predicted naturally occurring amino acid positions that correlate with antigenic changes among different FMDV SAT3 isolates, previously characterized by their *in vitro* cross-neutralizing capacity against a reference serum. The mechanisms of induction of innate immune responses by non-coding synthetic RNA mimicking structural domains in the FMDV genome was also analyzed by Rodriguez-Pulido et al. using a cell line derived from wild boar lung cells. In the same way, Medina et al. reviewed the use of interferon-based biotherapeutics to boost the innate immunity and block FMDV replication in natural hosts.

In close relation with the immunity induced in natural hosts after infection, fundamental studies on the FMDV pathogenesis are paramount to design new intervention strategies and evaluate actual risks related to managing and trading of animals and animal-derived products. Muthukrishnan et al. provided novel information on the FMD pathogenesis and humoral immune responses elicited in small ruminants after experimental infection with a serotype O virus strain. Zhu et al. presented new information on the differential expression in a set of bovine genes previously identified in transcriptomic studies performed on nasopharyngeal tissue samples from FMDV carriers and non-carriers. Viral persistence in cattle and buffalos was also investigated by Bertram et al. by developing statistical models to describe the extinction of FMDV persistent infection using data from primary longitudinal studies of naturally infected animals. A mathematical model is presented by Cabezas et al. to estimate the potential FMDV transmission in cattle, according to the livestock production methods in the US. Dekker et al. also used mathematical modeling, applied to previous *in vivo* experiments, to quantify the effect of vaccination and physical distancing on the FMDV transmission in pigs. Also working in swine, Stenfeldt et al. produced experimental data on infection associated with FMDV-infected pigs, showing results on the *in vivo* transmission between infected and naïve animals, and the persistence of FMDV infectivity in refrigerated carcasses.

This wide collection of original manuscripts undoubtedly contributes to the understanding of the disease and the development of suitable tools and methods for FMD control. However, there are still research gaps that need to be fulfilled to provide the knowledge and tools required to advance the progressive global control of FMD. It is noteworthy that no research papers related to vaccine selection and protection against heterologous FMDV strains were submitted. This subject, together with basic immunity, constitute one of the areas where there is still room for improvement. More work is needed to expand our understanding of vaccine cross-protection, especially in heterologous systems, information that is critical to select suitable vaccines to respond to FMD outbreaks and in the designing of antigen and vaccine banks.

The eradication of FMD is complex and must involve the integration of new approaches as control strategies. Successful health management policies to contain and eradicate FMD must combine diverse intervention and outbreak mitigation approaches. In this context, GFRA will continue promoting multidisciplinary scientific research among its partners aiming to develop comprehensive responses to the numerous challenges still posed by FMD at the global scale.

## Author Contributions

All authors listed have made a substantial, direct and intellectual contribution to the work, and approved it for publication.

## Conflict of Interest

The authors declare that the research was conducted in the absence of any commercial or financial relationships that could be construed as a potential conflict of interest.

## References

[1] Rushton J Knight-Jones TJ. The Impact of Foot and Mouth Disease. (2012). Available online at: http://www.oie.int/doc/ged/D11888.PDF (accessed April 2, 2021).

[2] Knight-Jones TJD McLaws M Rushton J. Foot-and-mouth disease impact on smallholders - what do we know, what don't we know and how can we find out more? Transbound Emerg Dis. (2017) 64:1079–94. 10.1111/tbed.12507 27167976 PMC5516236

[3] Knight-Jones TJ Rushton J. The economic impacts of foot and mouth disease - what are they, how big are they and where do they occur? Prev Vet Med. (2013) 112:161–73. 10.1016/j.prevetmed.2013.07.013 23958457 PMC3989032

[4] Sobrino F Saiz M Jimenez-Clavero MA Nunez JI Rosas MF Baranowski E. Foot-and-mouth disease virus: a long-known virus, but a current threat. Vet Res. (2001) 32:1–30. 10.1051/vetres:2001106 11254174

[5] Alexandersen S Mowat N. Foot-and-mouth disease: host range and pathogenesis. Curr Top Microbiol Immunol. (2005) 288:9–42. 10.1007/3-540-27109-0_2 15648173

